# Guided self-help interventions for mental health disorders in children with neurological conditions: study protocol for a pilot randomised controlled trial

**DOI:** 10.1186/s13063-016-1663-z

**Published:** 2016-11-04

**Authors:** Sophie Bennett, Isobel Heyman, Anna Coughtrey, Jess Simmonds, Sophia Varadkar, Terence Stephenson, Margaret DeJong, Roz Shafran

**Affiliations:** 1 UCL Institute of Child Health, 30 Guilford Street, London, WC1N 1EH UK; 2 Great Ormond Street Hospital for Children NHS Foundation Trust, Great Ormond Street Hospital, Great Ormond Street, London, WC1N 3JH UK

**Keywords:** Mental health disorders, Neurological conditions, Guided self-help, Cognitive behaviour therapy, Children

## Abstract

**Background:**

Rates of mental health disorders are significantly greater in children with physical illnesses than in physically well children. Children with neurological conditions, such as epilepsy, are known to have particularly high rates of mental health disorders. Despite this, mental health problems in children with neurological conditions have remained under-recognised and under-treated in clinical settings. Evidence-based guided self-help interventions are efficacious in reducing symptoms of mental health disorders in children, but their efficacy in reducing symptoms of common mental health disorders in children with neurological conditions has not been investigated. We aim to pilot a guided self-help intervention for the treatment of mental health disorders in children with neurological conditions.

**Methods/design:**

A pilot randomised controlled trial with 18 patients with neurological conditions and mental health disorders will be conducted. Participants attending specialist neurology clinics at a National UK Children’s Hospital will be randomised to receive guided self-help for common mental health disorders or to a 12-week waiting list control. Participants in the treatment group will receive 10 sessions of guided self-help delivered over the telephone. The waiting list control group will receive the intervention after a waiting period of 12 weeks. The primary outcome measure is reduction in symptoms of mental health disorders. Exclusion criteria are limited to those at significant risk of harm to self or others, the presence of primary mental health disorder other than anxiety, depression or disruptive behaviour (e.g. psychosis, eating disorder, obsessive-compulsive disorder) or intellectual disability at a level meaning potential participants would be unable to access the intervention. The study has ethical approval from the Camden and Islington NHS Research Ethics Committee, registration number 14.LO.1353. Results will be disseminated to patients, the wider public, clinicians and researchers through publication in journals and presentation at conferences.

**Discussion:**

This is the first study to investigate guided self-help interventions for mental health problems in children with neurological conditions, a group which is currently under-represented in mental health research. The intervention is modular and adapted from an empirically supported cognitive behavioural treatment. The generalisability and broad inclusion criteria are strengths but may also lead to some weaknesses.

**Trial registration:**

Current Controlled Trials: ISRCTN21184717. Registered on 25 September 2015.

**Electronic supplementary material:**

The online version of this article (doi:10.1186/s13063-016-1663-z) contains supplementary material, which is available to authorized users.

## Background

Around 15 % of young people have a long-term physical health condition [1]. Rates of mental health disorders are significantly greater in children with physical illnesses than in healthy controls [2, 3], and those with neurological conditions have up to a ninefold greater risk of emotional and behavioural disorders [4]. There are usually multiple reasons for the association between physical and mental health. Physical and mental health conditions may share the same underlying pathology, particularly in neurological conditions, such as epilepsy [4, 5]. Furthermore, children with physical illnesses face a number of challenges, such as maintaining a medication regime, being unable to participate in physical activity and feeling different or isolated from their peers [6]. This stress on the individual and his/her family [7] may put the child at greater risk of mental health disorders [8]. The relationship between epilepsy and behavioural and emotional symptoms is complex, as such symptoms may be affected by medications [9], surgery [10] and seizure location/activity. Children and young people with epilepsy are also more likely to have autism spectrum disorder [11], attention deficit hyperactivity disorder (ADHD) [12] and intellectual disability [13], conditions which themselves are associated with a greater risk of mental health disorders [14].

Not only does mental illness have considerable consequences for a child’s quality of life and behavioural/educational and social functioning [15, 16], but mental ill health has, in turn, also been shown to affect management and medical consequences of the physical illness. For example, mental illness has been linked to greater frequency of seizures in epilepsy [17].

There are numerous highly effective evidence-based interventions for mental health disorders in children and young people; one review found 750 treatment protocols for evidence-based treatments of mental health disorders in children [18]. The National Institute for Health and Care Excellence (NICE) recommends the use of psychological therapies for common mental health problems in children, e.g. group or individual parenting interventions for children with disruptive behaviour disorders [19] and cognitive behavioural therapy (CBT) for anxiety and depression [20, 21]. However, there is limited guidance on the use of these therapies in children with physical illness, and in many cases there remains a large unmet need. For example, Ott and colleagues [22] found that of 114 children with epilepsy, 61 % had psychiatric diagnoses, but, of these, only 33 % had received treatment, despite regularly attending clinics for their epilepsy. A recent review of the psychiatric aspects of childhood epilepsy [23] concluded that ‘the psychiatric problems in children with epilepsy have remained under-recognised and under-treated in clinical settings’. There is little existing research on the utility and efficacy of evidence-based therapies for children with mental health disorders and neurological conditions [24]. There is a need to improve the uptake of evidence-based therapies for children with mental health disorders and neurological conditions.

One way of meeting a large need for psychological therapy is through the use of guided self-help interventions. A recent meta-analysis of psychotherapy for depression and anxiety disorders in adults found that guided self-help interventions and face-to-face interventions are equally efficacious. A second systematic review demonstrated that the use of guided self-help interventions may be effective in adults with physical health problems [25], and such interventions including guided self-help and computerised CBT are recommended for adults with long-term physical health conditions and depression [26]. Guided self-help interventions have been shown to be beneficial in reducing symptoms of anxiety, depression and behavioural problems in children without physical illness [27–32]. Parent-delivered self-help interventions are appropriate for children aged 7–12 years old [29–31], whereas self-help conducted directly with the patient is recommended for those aged 13 and older [32, 33]. Guided self-help may be especially beneficial to people with physical illnesses, due to their potential difficulties in travelling to appointments. Additionally, children with neurological conditions are likely to have multiple medical appointments and must also attend school, and scheduling a time to meet with a therapist may be difficult in this context.

There has been no research to date examining the efficacy of self-help interventions in reducing symptoms of common mental health disorders in children with neurological conditions. The aim of this research is to conduct a pilot study to investigate the efficacy of guided self-help for symptoms of common mental health disorders in children with neurological conditions, in a preliminary randomised controlled trial (RCT) design. Compared to a waiting list control, we anticipate that children who receive guided self-help will demonstrate clinically significant reductions in anxiety, depression and disruptive behaviour, through parent-report measures (primary outcome measures). We also predict that following completion of guided self-help, children will demonstrate improvements in quality of life, also through parent-report measures.

### Aims

The study aims to pilot an intervention in a group of attendees at a tertiary paediatric neurology service. As a pilot RCT, the main aim of this study is to inform a future powered RCT. We will investigate whether the components of the study work effectively together, estimate recruitment and retention rates and determine the expected effect size for the primary outcome measure [34]. We will also aim to investigate acceptability to young people and families of both the study design (e.g. randomisation, measures, etc.) and the intervention for impairing symptoms of anxiety, depression and disruptive behaviour disorders.

## Methods/design

### Study design

**Fig. 1:**
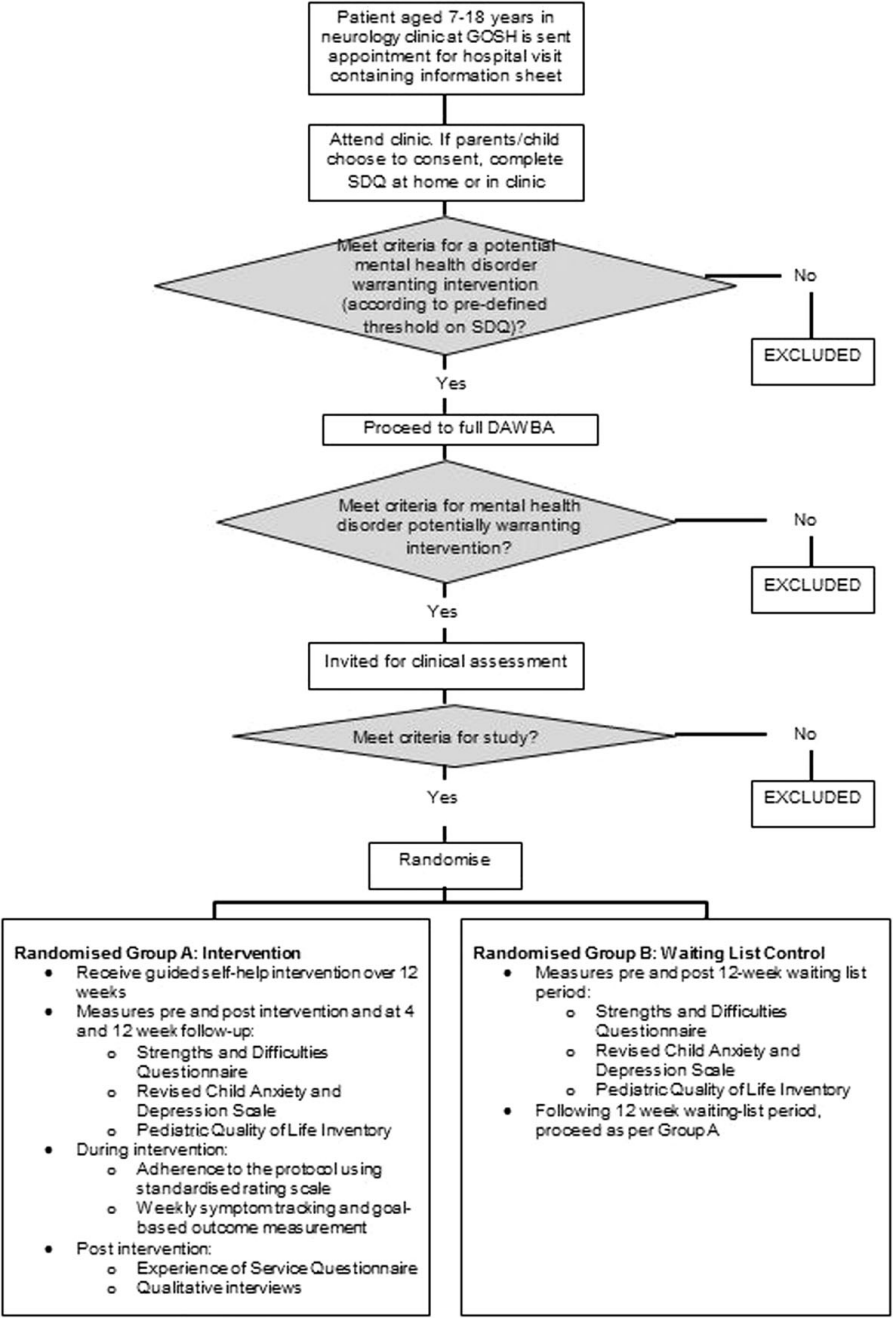
Flow chart of study process

The study is a pilot RCT conducted at Great Ormond Street Hospital (GOSH), UK. Eligible children attending a neurology clinic and who have impairing symptoms of common mental health disorders (anxiety, depression and/or disruptive behaviour) will be randomly allocated to either (1) receive guided self-help for their mental health disorder over 12 weeks or (2) remain on a waiting list for 12 weeks with no additional intervention. Children randomised to the waiting list condition will be offered the guided self-help intervention at the end of 12 weeks. All participants will complete follow-up assessments at 4 and 12 weeks post-intervention (Fig. 1). Methods are in accordance with the Standard Protocol Items: Recommendations for Interventional Trials (SPIRIT) statement (Additional file 1).

### Population and recruitment process

The sample will consist of children aged 7–18 years attending a neurology clinic waiting for/undergoing assessment or treatment at GOSH, who are identified as meeting criteria for impairing symptoms of anxiety, depression or disruptive behaviour. Children will be recruited across all neurology clinics at GOSH with the exception of migraine, neurodisability and multiple sclerosis clinics for practical reasons. The children will therefore have a range of neurological conditions, including epilepsy, cerebrovascular conditions and brain tumours. Inclusion and exclusion criteria are listed in Table 1. It is possible that children and young people attending neurology clinics may in fact be in the process of obtaining a diagnosis and may have no underlying neurological condition. As a tertiary centre, many patients may come to GOSH due to a complex presentation where the diagnosis is unclear. Most patients attending neurology clinics at GOSH have diagnoses of epilepsy. However, among these patients, some with a diagnosis of epilepsy may also have non-epileptic seizures (in combination with epileptic seizures or separately). Given this significant overlap, and as the purpose of this study is to widen access to therapy, we did not want to impose an arbitrary inclusion/exclusion criterion of a diagnosed neurological condition, so that treatment would not be delayed for those who could potentially benefit.

**Table 1:** Inclusion and exclusion criteria

Inclusion criteria
1. Young people aged 7–18 years. 2. Waiting for/undergoing assessment or treatment in a neurology clinic at Great Ormond Street Hospital. 3. Identified as meeting criteria for symptoms of common mental health disorders (i.e. anxiety, depression or behavioural problems).
Exclusion criteria
1. Children/families who do not speak/understand English well enough to access the screening assessments and interventions. 2. Children who have a learning disability at a level meaning that they cannot access the screening and/or intervention. This will be determined by clinical judgement, either prior to recruitment, if the child is already known to GOSH, or at the initial assessment for guided self-help. This will not be defined by IQ, but by clinical judgement; children will not be excluded because they have a learning disability per se, but because they are unable to access the materials. Ability to participate may be different for younger children, whose parents complete the materials, compared to older children, who need to complete the materials themselves. 3. Children who screen for a severe mental health disorder other than depression, anxiety or disruptive behavioural disorders or whose screening is otherwise suggestive of risk will be excluded and referred to other services as appropriate.

Information leaflets will be sent out with clinic letters to all children aged 7–18 attending neurology clinics at GOSH. Notices will be posted in the clinic waiting rooms, informing patients of the project and providing contact details. A researcher will be present at the clinic and will spend at least 15 minutes with each child/family, taking time to further explain the study and answer any questions that potential participants may have. The researcher will obtain informed consent/assent at this point, for those children and families who agree to participate.

### Sample size

One of the primary aims of this pilot trial is to estimate the effect size to inform a future full-scale trial. Sample size is therefore estimated from the expected recruitment rate and thus the possible number that can be recruited in one year (the length of the study).

There are 5195 neurology clinic sessions with 2755 patient referrals at GOSH per annum, of which we estimate that two-thirds will fall within the correct age range as patients are seen routinely throughout their childhood (*n* = 1837), and based on clinic data, 15 % will cancel/not attend appointments (*n* = 1561). Of those attending, around 25 % will have an intellectual disability that will mean they are excluded according to criterion 2 in Table 1 [29], leaving *n* = 1170. Assuming 50 % agree to participate (average screening consent rate from Bennett et al., 2015 [24]), we aim to consent 585 participants to the initial screening.

Based on population rates, we conservatively estimate that 10 % of those screened will have a mental health condition warranting intervention (i.e. 59; [35]). Estimating that 66 % will complete the full Development and Well-Being Assessment (DAWBA) [36], around 39 will be potentially eligible for the full intervention. Of these, 40 % (16) may already be receiving input from mental health services [22], leaving 24 young people who are potentially eligible. We estimate that 75 % of these will agree to participate (*n* = 18 children and young people; the mean agreement for intervention rate from Bennett et al., 2015 [24]).

We will only recruit participants whilst there is capacity for intervention. If there is a point at which capacity is almost reached, recruitment and screening will be suspended accordingly. In order to estimate capacity, we will determine a priori how much time each therapist can commit and therefore how many patients they can support per week.

### Intervention

#### Guided self-help arm

The guided self-help is based on the Modular Approach to Therapy for Children with Anxiety, Depression, Trauma, or Conduct Problems (MATCH-ADTC) [37]. This combines modules for the treatment of anxiety, depression and behaviour problems, taken from known evidence-based protocols, with an empirically derived algorithm for making decisions regarding which module should be used and when. It is particularly suitable for populations with significant comorbidity, as is seen in young people with epilepsy and other neurological conditions. The main techniques that will be used are parent-delivered CBT for anxiety, problem solving for depression and parent training approaches for externalising difficulties. The adapted treatment package has been piloted with six children and young people with co-occurring mental health problems and neurological disorders. The treatment package has been revised and updated based on the findings of this pilot data and from feedback from children, young people and their families. For example, following feedback from families that one of the worksheets in the section on intervention for behavioural difficulties was confusing, this worksheet was removed from the self-help materials.

In line with previous research, the guided self-help will take the format of one face-to-face assessment and 10 telephone sessions over 12 weeks.

#### Waiting list control arm

Children allocated to remain on the waiting list will be offered the guided self-help intervention after 12 weeks. Participants on the waiting list will be given details of whom to contact in an emergency (and how to recognise signs of risk) should the symptoms of mental health disorders become significantly worse during this time period. This is in accordance with usual care. Children will be fast-tracked to appropriate services should significant risk be identified in this time period, in accordance with best practice guidance.

### Training, supervision and treatment adherence

Supporters assisting the guided self-help will be trained to deliver the treatment protocol. All therapists will receive weekly supervision from an experienced clinical psychologist. Adherence to the treatment protocol will be measured using a standardised rating scale [25].

### Outcome measures

#### Strengths and Difficulties Questionnaire

The Strengths and Difficulties Questionnaire (SDQ) [38] is a 25-item psychometrically robust questionnaire used for the identification of common mental and behavioural symptoms in young people. The SDQ is one of the most widely used measures of childhood psychopathology in the UK. It has been chosen as the primary outcome measure since it measures depression, anxiety and behavioural difficulties and can therefore be used by the entire sample. It has excellent reliability and validity [38].

#### Development and Well-Being Assessment

The DAWBA [39] is a computerised clinical assessment which generates ICD-10/DSM-5 psychiatric diagnoses. Parent measures alone are completed for children aged 10 and younger. Parent and child measures are completed for those aged 11 and over. The SDQ forms part of the DAWBA.

#### Revised Children’s Anxiety and Depression Scale

The Revised Children’s Anxiety and Depression Scale (RCADS) [40] is a 47-item questionnaire with robust psychometric properties used to assess depression and anxiety in children and young people.

#### Pediatric Quality of Life Inventory Generic Core Scales

The emotional functioning (five items) and physical functioning (eight items) subscales of the Pediatric Quality of Life Inventory (PedsQL) [41] will be used to examine quality of life. The PedsQL has excellent reliability and validity [35]. There are different versions of the PedsQL for different age ranges (young children aged 5–7; children aged 8–12; teens aged 13–18). The appropriate questionnaire for the age of the child will be used.

Additionally, we will record each participant’s IQ. Relevant medical details will be recorded, including specific neurological diagnoses, duration of neurological symptoms, medication or other relevant medical interventions at assessment, pre-intervention/post-waiting list, post-intervention and follow-up time points. These variables will be examined as potential moderators of outcome.

### Assessments

Assessment will be at screening, baseline, end of waiting list (for those in the waiting list control arm), end of treatment and at follow-up at 4 and 12 weeks post-intervention.

#### Screening and eligibility assessment

All children meeting inclusion criteria and who consent to participate will be screened using the computerised SDQ before completing the full DAWBA. The computerised SDQ and DAWBA will be completed either at home or in the neurology clinics at GOSH, depending on the patient/family’s preference.

#### Baseline assessment (plus post-waiting list assessment for those allocated to waiting list control)

All families in the intervention arm will complete a clinical assessment. This will be a face-to-face assessment with the family (or young person alone if appropriate), completed by a qualified clinical psychologist. The child’s paediatrician or other members of their care team will be consulted as necessary. Paper copies of the RCADS and PedsQL will be completed at this point.

#### End-oftreatment assessment

End-of-treatment assessment will include completion of endpoint measures (DAWBA incorporating SDQ, PedsQL, RCADS and Experience of Service Questionnaire).

#### Follow-up assessments

Follow-up assessments will include completion of follow-up measures at 4 and 12 weeks after completion of the intervention (SDQ, PedsQL and RCADS).

### Session-by-session measures

Families will also complete session-by-session outcome measurements, in accordance with the Children and Young People’s Improving Access to Psychological Therapies (CYP-IAPT) data specification [42]; these will include a brief symptom-specific measure and goal-based outcome rating.

### Randomisation, blinding and code-breaking

Participants identified by the DAWBA and clinical assessment as experiencing impairing symptoms of mental health disorder and neurological conditions will be randomised to (1) guided self-help for their mental illness over 12 weeks or (2) remaining on the waiting list for guided self-help with no additional intervention over 12 weeks. Stratified by type of mental health disorder (anxiety, depression or disruptive behaviour), participants will be randomised 1:1 to the treatment arms using random blocks. An independent researcher will provide the randomisation codes, hold them and break the code following completion of the trial.

### Adverse events

All adverse events observed by the study team or reported by participants will be reported to the ethics committee. In the event that significant risk or a mental health difficulty that is not appropriate for guided self-help is detected, through any phase of the treatment, participants will be referred to other services as appropriate. This may include referral to local child and adolescent mental health services, general practitioner, GOSH mental health services or paediatric psychology, for example. The study team may also contact the duty clinician where appropriate if immediate action is required. The study team will arrange for follow-up appointments or telephone calls until the risk is adequately managed, consistent with best practice guidance [43]. If the intervention is not successful and the child is in need of further, higher intensity treatment, then a referral will be made to the appropriate service.

All serious adverse events will be reported in accordance with Good Clinical Practice guidelines and local policies, including being reported to the local research and development team and ethics committee within 24 hours of the study team becoming aware of the event.

### Statistical analysis

#### Primary endpoint efficacy analysis

As a pilot, the study is not powered to detect a statistically significant difference between the intervention and waiting list groups. Instead, we will use the results to estimate the effect size of the difference in order to inform a power calculation for a planned full-scale RCT. All analyses will be carried out on an intention-to-treat basis and will be blind to treatment assignment. Treatment groups will be descriptively compared on all baseline measures. No interim analyses are planned, but outcome at the end of treatment will be evaluated prior to data lock for follow-up data. The primary analysis will use the end-of-treatment SDQ assessment to compare the intervention and waiting list control groups. A linear regression adjusting for baseline assessment of the SDQ and other baseline measures for which imbalance is evident will be used to evaluate the intervention effect. Whilst we recognise that the SDQ is not as sensitive at detecting change as other measures such as the RCADS, it is the only measure which is able to detect change across all of the symptoms addressed within the study (anxiety, depression and behavioural difficulties). As diagnosis may be an important moderator of treatment outcome, we will conduct sensitivity analyses by analysing young people with confirmed epilepsy diagnoses (assumed to be the largest subset of participants) separately.

#### Follow-up data

Outcome analyses will follow the pattern of those of the primary endpoint. Outcomes will be reported in accordance with the Consolidated Standards of Reporting Trials (CONSORT) statement.

### Collection of feasibility data

In order to inform a larger trial, we will collect data on:

- Recruitment rates
- Data completion rates
- Intervention completion rates
- Average length of support sessions
- Acceptability of the intervention
- Time taken to complete measures

### Qualitative analysis

We will interview children and young people and their families and clinicians about their experiences of the study and the intervention, including suggestions for improvement. These data will be analysed thematically.

## Ethics and dissemination

### Ethical approval

This study has been reviewed and approved by the Camden and Islington Research Ethics Committee (registration number 14.LO.1353). All participants will receive verbal and written information about the study, and all patients must give written informed consent before inclusion and randomisation. The study has been registered with the Current Controlled Trials register (ISRCTN21184717).

### Informed consent

Participants will be informed that their participation is voluntary and that they can withdraw at any time without providing a reason and without their medical care or legal rights being affected. Participants will be given the opportunity to ask any questions, and only when all of their queries have been answered will consent/assent be taken by the principal investigator or a suitably qualified delegated member of the research team. The participant’s informed consent will be obtained from all parents and/or carers of children taking part in the study. Where possible, older children and young people will provide informed consent and younger children will provide informed assent.

### Ethical and safety considerations

It is possible that the questionnaires, assessment and intervention may cause participants and/or carers to become distressed. The research team is made up of a number of clinically trained researchers, who will be well-placed to manage this distress should it arise and to signpost to sources of support (such as local mental health services) if necessary. Some participants will be randomised to remaining on the waiting list. However, all children will eventually receive the guided self-help intervention. We are not depriving any children of an intervention that they would otherwise have received.

### Patient confidentiality and data protection

Patient identifiable data, including initials, date of birth and NHS number will be required for the registration process. The study team will ensure that the participants’ anonymity is maintained. The participants will be identified only by initials and a participant ID number on the case report form (CRF) and any electronic database. All documents will be stored securely and will be accessible only by study staff and authorised personnel. The study will comply with the Data Protection Act, which requires data to be anonymised as soon as it is practical to do so.

### Dissemination plan

During the study, a public website will be launched and made available to participants and the health care professionals involved. It will include information and updates about the progress of the study. The results will be published in peer-reviewed scientific journals and will be disseminated at relevant research conferences and via internal reports. Written feedback will be provided to patient support groups.

## Discussion

This is the first study to investigate guided self-help interventions for mental health problems in children with neurological conditions — a group which is currently under-represented in mental health research. The intervention is modular and adapted from an empirically supported cognitive behavioural treatment. The generalisability and broad inclusion criteria are a strength but may also lead to some weaknesses, including the possibility of a child not having a confirmed neurological diagnosis and no standardised measure of physical health. In addition, this initial study will be based within a specialist hospital, which may limit generalisability. However, it also has a number of strengths, including random allocation and blinding of study assessors, enhancing internal validity of the results. It aims to be as generalisable to routine clinical practice as possible and has minimal exclusion criteria. In addition, the outcome measures used are those used in UK National Child and Young Persons Improving Access to Psychological Therapies (CYP-IAPT) services, allowing results to be compared to those across Child and Adolescent Mental Health (CAMH) services nationally.

We anticipate the study will yield important information on the feasibility of a large-scale study to assess the clinical and cost-effectiveness of guided self-help for common mental health disorders in the context of neurological illness.

## Trial status

Recruitment began in January 2015 and recruitment to the study is due to end in January 2016 with all participants completing follow-up by August 2016.

## Additional file

Additional file 1: SPIRIT 2013 Checklist: Recommended items to address in a clinical trial protocol and related documents*. (DOC 122 kb)
